# PGC‐1α protects from myocardial ischaemia‐reperfusion injury by regulating mitonuclear communication

**DOI:** 10.1111/jcmm.16236

**Published:** 2021-01-19

**Authors:** Yan‐Qing Li, Yan Jiao, Ya‐Nan Liu, Jia‐ying Fu, Lian‐Kun Sun, Jing Su

**Affiliations:** ^1^ Department of Pathophysiology College of Basic Medical Sciences Jilin University Changchun China; ^2^ Department of Hepatobiliary and Pancreatic Surgery The First Hospital of Jilin University Changchun China

**Keywords:** mitochondria, myocardial ischaemia‐reperfusion injury (IRI), nuclear factor, erythroid 2 like 1/2 (NRF‐1/2), nucleus, oxidative stress, peroxisome proliferator‐activated receptor gamma co‐activator‐1α (PGC‐1α)

## Abstract

The recovery of blood supply after a period of myocardial ischaemia does not restore the heart function and instead results in a serious dysfunction called myocardial ischaemia‐reperfusion injury (IRI), which involves several complex pathophysiological processes. Mitochondria have a wide range of functions in maintaining the cellular energy supply, cell signalling and programmed cell death. When mitochondrial function is insufficient or disordered, it may have adverse effects on myocardial ischaemia‐reperfusion and therefore mitochondrial dysfunction caused by oxidative stress a core molecular mechanism of IRI. Peroxisome proliferator‐activated receptor gamma co‐activator 1α (PGC‐1α) is an important antioxidant molecule found in mitochondria. However, its role in IRI has not yet been systematically summarized. In this review, we speculate the role of PGC‐1α as a key regulator of mitonuclear communication, which may interacts with nuclear factor, erythroid 2 like ‐1 and ‐2 (NRF‐1/2) to inhibit mitochondrial oxidative stress, promote the clearance of damaged mitochondria, enhance mitochondrial biogenesis, and reduce the burden of IRI.

## 1 INTRODUCTION

Cardiovascular diseases are key medical issues with frequently fatal outcomes. According to statistics of the World Health Organization, about one sixth of the global mortality annually is caused by myocardial ischaemia‐reperfusion injury (IRI).^1^ In the past decades, researchers have found several therapeutic approaches that can prevent and reduce IRI, such as calcium channel blockers, anti‐inflammatory drugs, erythropoietin, atorvastatin, adenosine and anti‐hyperglycaemic agents.^2^, ^3^, ^4^ However, limited by their inherent characteristics, these therapeutics have so far remained largely theoretical and not yet achieved satisfactory clinical results. Therefore, there is an urgent need to find more suitable and clinically actionable treatments for IRI.

Peroxisome proliferator‐activated receptor gamma co‐activator‐1α (PGC‐1α) is one of the most studied nuclear cofactor family members. As an auxiliary regulator, it plays an important role in polyprotein complexes. On one hand, it binds transcription factors binding to DNA sequences in complexes; on the other hand, it can interact with some nuclear factors, such as nuclear factor, erythroid 2 like‐1 (NRF‐1) and −2 (NRF‐2).^5^, ^6^, ^7^, ^8^ In addition, previous studies have found that overexpression of PGC‐1α helps to reduce myocardial ischaemia. Therefore, PGC‐1α may be a potential therapeutic target for IRI.

Mitochondria mainly exist in the myocardium, which has a high energy demand.^10^ Here, they do not only produce large amounts of adenosine triphosphate via oxidative phosphorylation in order to maintain the normal physiological function of cells, but also participate in the cell matrix metabolism, signal transduction pathways, and other cellular activities.^11^ In order to maintain the mitochondrial function stability, mitochondria participate in the anterograde regulation of the nucleus, as well as produce a retrograde response to regulate nuclear gene expression.^12^, ^13^, ^14^ This mitonuclear communication by positive regulation and reverse feedback constitutes a functional interaction which helps to maintain mitochondrial function and can protect cells from external damage.^15^

This article reviews the cardioprotective effects of PGC‐1α in IRI. Firstly, the structure, expression, and location of PGC‐1α are described to provide an overview of PGC‐1α. In addition, we summarized the importance of mitonuclear communication in the myocardium. At the same time, the potential protective effects of PGC‐1α on IRI via mitonuclear communication were described in detail. Finally, the therapeutic potential and future research directions involving PGC‐1α in IRI are presented.

## 2 OVERVIEW OF PGC‐1α

### 2.1 Structural characteristics of PGC‐1α

**FIGURE 1:**
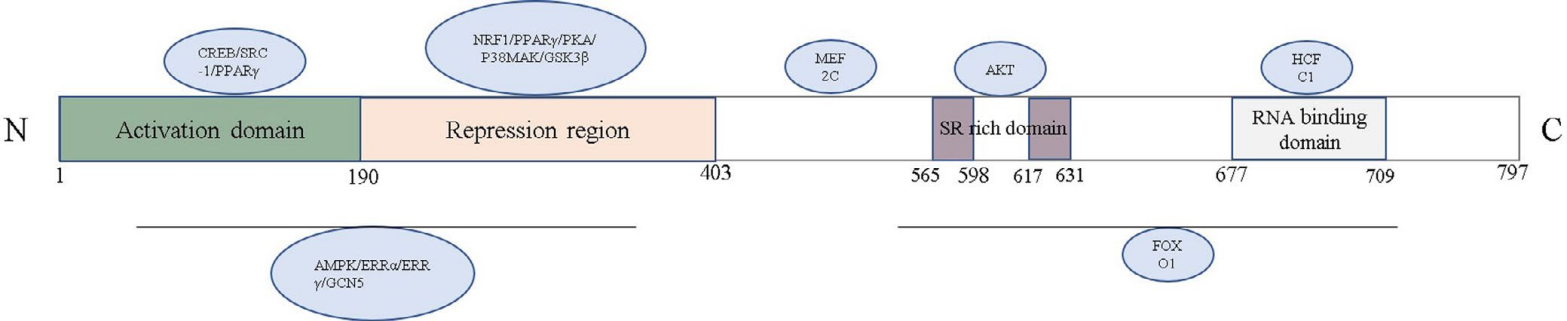
Functional domains of PGC‐1α. A schematic representation of PGC‐1α functional domains involved in the regulation of IRI

The PGC‐1α gene is highly conserved and located on the reverse strand of chromosome 4 (23, 755, 041‐23, 904, 089).^16^ There are 18 transcripts (splice variants), 182 lineage homologues and 2 lineage homologues of PGC‐1α, and it shares similar domain structures with other nuclear cofactor family members. The protein contains three functional domains, including an N‐terminal activation domain, a central regulatory domain, and a C‐terminal ribonucleic acid binding domain.^17^ The N‐terminal activation domain consists of a transcriptional activation domain and a leucine‐rich LXXLL motif, which is a key motif for nuclear receptor interaction.^18^ The C‐terminal ribonucleic acid binding domain consists of a ribonucleic acid recognition and binding site (RRM, ribonucleic acid recognition motif) and a serine‐ and arginine‐rich structural domain (RS, serine/arginine enrichment domain).^19^ As the PGC‐1α family lacks enzymatic activity, PGC‐1α acts as an anchoring platform for other proteins with histone acetyltransferase activity, and triggers gene transcription by promoting the assembly of transcription mechanisms, thus playing a regulatory role in gene transcription (Figure 1). PGC‐1 α promoter contains a common sequence (ARE) are binding to NRF. NRF is likely to interact with the N‐terminal activation domain, C‐terminal SR and RNA binding domain of PGC‐1 α.

### 2.2 **Expression and localization of PGC‐1**α

Since PGC‐1α was discovered to contribute to heat production, specifically in brown adipose tissue, in the 1990s,^20^ many studies on PGC‐1α have accumulated. PGC‐1α typically is located in the cytosol and nucleus, where it performs its biological functions under physiological and pathological conditions. PGC‐1α is highly expressed in metabolically active tissues, such as brown adipose tissue, heart, kidney, skeletal muscle, and brain.^21^ In addition, the expression of PGC‐1α can be further induced under various stress conditions. For example, PGC‐1α is activated by nutritional deprivation, oxidative damage, and chemotherapy.^22^ Moreover, high expression levels of PGC‐1α have been found to be a critical protective factor in IRI.^23^, ^24^

## 3 ROLE OF MITONUCLEAR COMMUNICATION IN IRI

### 3.1 Mitochondrial dysfunction during IRI

**FIGURE 2:**
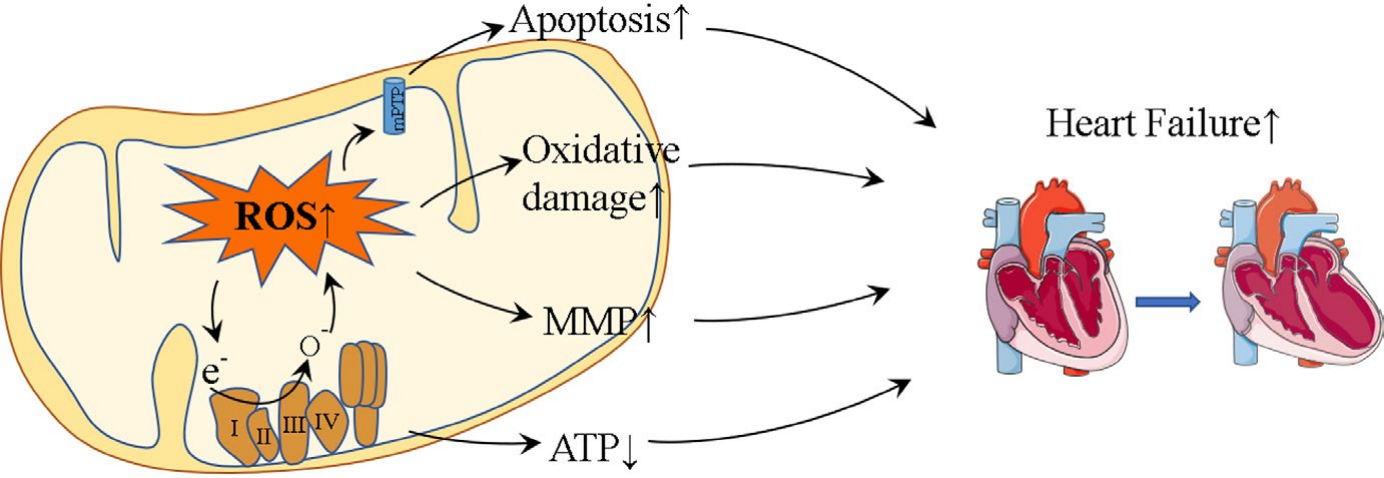
Role of mitochondria in IRI. In IRI, mitochondria attacked by ROS in turn produce increased levels of ROS, which can exacerbate damage. In addition, ROS lead to a decrease of ATP levels, oxidative damage, MMP and apoptosis, which eventually leads to heart failure

Mitochondria are the key producers of energy in eukaryotic cells. The number of mitochondria varies in different tissues and cells based on varying amounts of energy demand.^25^ Cardiac muscle cells are highly metabolically active and energy‐demanding and therefore have a high number of mitochondria, which account for 40% of the cell volume.^26^ During myocardial ischaemia, an increase in reactive oxygen species (ROS) in the myocardial submucosal matrix and an inhibition of mitochondrial permeability transition pore (mPTP) opening are observed. The opening of mPTP and oxidative stress caused by ROS are considered the main mechanisms of mitochondrial and myocardial dysfunction.^27^ In addition, mitochondria in myocardial fibrous matrix decrease the activity of aconitase, an enzyme of the tricarboxylic acid cycle, and have no inhibitory effect on the production of ROS and the opening of mPTP.^22^ Compared with the cardiac fibrous matrix, the cardiac submucosal matrix has a protective effect on mitochondria, which is related to the reduction of ROS production by inhibition of the activity of the mitochondrial electron transfer chain complex.^28^ When rats were deprived of oxygen for a long time, the production of mitochondrial ROS decreased, the opening of mPTP was inhibited, and the apoptosis of subcellular stromal cells decreased.^29^ Therefore, cardiomyocytes have been thought to be beneficial to mitochondria in hypoxic environments. However, during reperfusion, tissues receive blood supply again, which greatly increases the number of free radicals, including superoxide anions, hydrogen peroxide and hydroxyl groups, resulting in an aggravated injury.^26^, ^30^ Free radicals are highly reactive and they can attack mitochondria in myocardial tissue, leading to mitochondrial dysfunction. However, mitochondria are not only a main target of ROS damage, but also an important source of ROS.^31^ ROS are predominantly produced by electron transfer chain complexes I and III. Concurrently, ROS production in mitochondria destroys the structure of mitochondrial membranes, resulting in the release of cytochrome C, thus triggering apoptosis via the mitochondrial pathway. It has been reported that mitochondria have a positive feedback regulatory role in the initial ROS production by increasing their own ROS levels, which is called ROS‐induced ROS release (RIRR).^32^ ROS produced by the RIRR pathway lead to opening of mPTP, Ca^2+^ overload, and the collapse of the mitochondrial membrane potential, thus further aggravating IRI. These changes can lead to reperfusion arrhythmia, myocardial stunning, apoptosis and necrosis, resulting in microvascular and macrovascular injuries.^33^ Several drugs target mitochondria in order to protect myocardial cells from IRI by reducing the levels of ROS, for example the peptide bendavia. Myocardial cells treated with bendavia exhibit higher survival rates than those of the control group.^34^ This protective effect is achieved by reducing ROS‐induced cell death during reperfusion and maintaining the mitochondria transmembrane potential (ΔΨm). Singer et al have found that ammonium tetrathiomolybdate (ATTM) significantly reduced the infarct size after myocardial ischaemia or cerebral ischaemia by reducing mitochondrial ROS, thus improving the survival rate of mice by preventing a severe haemorrhage.^35^ Although these drugs have achieved good efficacy in animals, they may have side effects in humans, so clinical experiments must be conducted to test their efficacy (Figure 2).

### 3.2 Mitonuclear communication during IRI

Eukaryote cell structures are the result of the co‐evolution of mitochondrial and nuclear genomes.^36^ Therefore, it is conceivable that this cooperation resulted in genes which are adapted to environmental challenges via nuclear‐mitochondrial genetic interactions. Among the 1200 proteins in mitochondria, 13 are encoded by mitochondrial genes (mtDNA),^37^ which account for a majority of the mitochondrial electron transfer chain complex.^38^ As only a small fraction of mitochondrial proteins is encoded by mtDNA genes, the nucleus and mitochondria must continuously coordinate the transcription and translation of mitochondrial proteins, as well as translocations and imports. KJ et al ^39^ have investigated this by using mitochondrial‐nuclear eXchange mice, in which the nuclear and mitochondrial genomes were exchanged among different murine strains. They showed that the combination of the nuclear‐mitochondrial genetic backgrounds significantly changed metabolic efficiency and body composition. Mendelian genetics and mitochondrial genetics do not unilaterally control gene expression and as a result, when mitochondria are stressed, the nucleus reacts accordingly.

PGC‐1α plays a key role in nuclear‐mitochondrial crosstalk. Mitochondria are not only the core production site of cellular energy, but also the regulator of many cellular functions such as the metabolism and apoptosis.^40^ Moreover, the function of mitochondria is tightly controlled by the nucleus, which can reduce or increase the activity of mitochondria and promote the biogenesis of mitochondria, depending on the bioenergetic need of the cell.^41^ For example, the nucleus can regulate the transcription of mitochondrial DNA and mitochondrial regulatory genes, such as AMP‐activated catalytic subunit alpha 1 (AMPK), sirtuin 1 (Sirt1), and PGC‐1α.^42^, ^43^ Conversely, mitochondria can generate a ‘retrograde regulation’ to send signals to the nucleus and change the expression of nuclear genes, thus changing the function of cells and re‐planning cellular metabolism.^44^ This response can be triggered by decreases in ATP levels, an increase in ROS or the release of mitochondrial Ca^2+^.^45^ Low levels of ATP activate AMPK and PGC‐1α, which regulate mitochondrial biogenesis and mitochondrial homeostasis. The increase of ROS and Ca^2+^ also activates AMPK, PGC‐1 α, and c‐Jun N‐terminal kinase (JNK) pathways via anterograde regulation.^46^

## 4 PGC‐1Α PROTECTS FROM IRI THROUGH ANTEROGRADE REGULATION OF MITONUCLEAR COMMUNICATION

**FIGURE 3:**
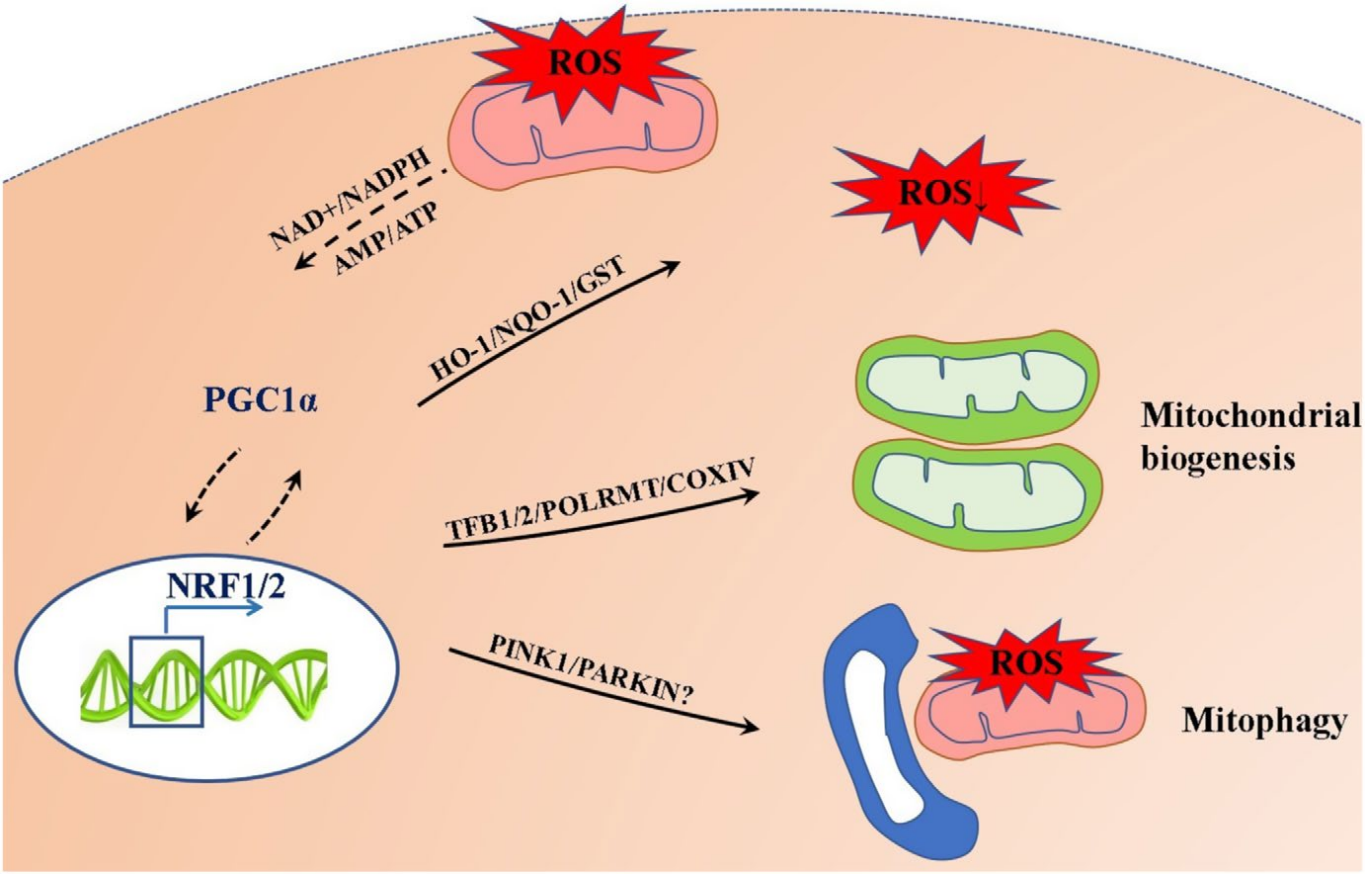
Role of PGC‐1α in IRI. Proposed model for the PGC‐1α‐NRF‐1/NRF‐2 signalling pathway in IRI. Mitochondrial dysfunction caused by myocardial ischaemia‐reperfusion injury leads to cell death. We illustrate how the PGC‐1α‐NRF‐1/NRF‐2 signalling pathway may orchestrate protection from mitochondria damage

During IRI, the function of mitochondria is compromised due to an increase of ROS. Clearing ROS and injured mitochondria, and generating new mitochondria is critical for myocardial cells in order to maintain their normal functions.^47^ We speculate that PGC‐1α actively regulates downstream factors through the mitonuclear communication during IRI, and ensures mitochondrial homeostasis by reducing ROS, which activates mitochondrial biogenesis and mitophagy (Figure 3).

### 4.1 ROS clearance pathway

NRF‐1 is closely associated with mitochondrial oxidative stress. Under oxidative stress, NRF‐1 binds to small Maf (sMaf) to form the NRF1‐MAFG heterodimer, which binds preferentially to the antioxidant response element sequence (ARE).^48^ In addition, the knockdown of NRF‐1 inhibits the expression of antioxidant genes encoding glutamate‐cysteine ligase catalytic subunit (GCLC), glutathione peroxidase 1 (GP1) and metallothionein 2 (MT‐2) in MC3T3‐E1 cells treated with lipopolysaccharide.^49^ These findings are consistent with the results of targeted NRF‐1 knockout in a severe oxidative stress model of mouse fibroblasts.^50^ What' more, the absence of NRF‐1 was not compensated by the presence of NRF‐2, which indicates that NRF‐1 plays a key role in antioxidant stress.^51^, ^52^, ^53^ Therefore, PGC‐1α may play a protective role in IRI by binding and co‐activating NRF‐1 and its downstream antioxidant genes.

NRF‐2 binds to ARE elements and transactivates a group of protective enzymes, such as haem oxygenase 1 (HO‐1), gamma‐glutamyl cysteine synthase (gamma‐GCS), peroxidase 1 (PRDX1), glutathione reductase (GR), and thioredoxin (SRXN).^54^ Drug metabolism and detoxification enzymes NADH quinone dehydrogenase 1 (NQO1), glutathione‐S‐transferase (GST), UDP‐glucuronosyltransferase and glucose‐6‐phosphate dehydrogenase have been used to alleviate oxidative stress injury.^55^, ^56^, ^57^ In addition, ARE‐like elements present in the promoter of NRF‐2 enhance its own transcription.^58^ Previous studies have shown that NRF‐2 activates downstream antioxidant genes during IRI, which significantly reduce myocardial injury.^51^, ^59^

Similar to NRF‐1, NRF‐2 also activates ARE elements (sMaF) by binding to sMaf. Under normal conditions, sMaf is a heteromeric chaperone of NRF‐1. However, under oxidative stress, NRF‐2 recruits sMaf proteins and deprives NRF‐1 of sMaf.^60^ This indicates that NRF‐2 may play a major role in the clearance of ROS. In addition, the absence of NRF‐1 is not compensated for by the presence of NRF‐2, which indicates that NRF‐1 plays an important role in coping with cellular stress. In both young and old mice, NRF‐2 knockout did not increase the compensatory ability of NRF‐1, but decreased the expression of NRF‐1.^61^ It has been found that NRF‐2 knockout reduces both the transcription of NRF‐1 and the downstream mitochondrial transcription factor A (mtTFA) in ROS as well as the nitric oxide (NO)‐induced adaptive responses of mouse skeletal muscle cells to exercise.^62^ However, NRF2 knockout mice were used in the experiment, and it is necessary to verify the interaction mechanism between NRF‐1 and NRF‐2 at the cellular level. From these studies, we speculated that NRF‐2 promotes the stability of NRF‐1 in complex networks.

ROS is also the early inducement of metabolic syndrome. Metabolic syndrome refers to the simultaneous existence of risk factors for atherosclerosis in an individual, including hyperglycaemia, dyslipidemia and hypertension. For example, in rats with metabolic syndrome induced by high fat and high sugar diet, excessive production of ROS is accompanied.^63^ Rats with large amounts of ROS have earlier insulin resistance, hyperlipidemia, and hypertension.^64^ Patients with metabolic syndrome may aggravate myocardial ischaemia‐reperfusion injury. PGC‐1α is the main way to eliminate ROS, which means that targeting PGC‐1α can eliminate ROS, reduce the occurrence of metabolic syndrome and ensure the health of the heart.

### 4.2 Mitochondrial biogenesis

Mitochondrial biogenesis refers to the formation of new mitochondria and their ability to produce ATP.^65^ Mitochondria have relatively independent genetic systems and biosynthetic sites.^66^ However, mitochondrial proliferation is a complex process that depends on the regulation of nuclear coding genes, with PGC‐1α being an important regulator of mitochondrial proliferation. PGC‐1α stimulates mitochondrial proliferation by activating transcription factors. It has been proved that NRF can bind to are sequence in promoter ARE region of mitochondrial biogenesis related genes (such as nuclear respiratory factor 1/2, Nrf1/2) to stimulate mitochondrial synthesis.^67^

Nrf1 activates the expression of nuclear genes essential for mitochondrial biogenesis and function, including key genes of the mitochondrial respiratory complex subunits, haem biosynthetase and regulatory factors involved in mtDNA replication and transcription.^68^ In addition, Nrf1 activates TFAM and transcription factor B1/2 (TFB1M/2M) to promote the transcription of mitochondrial genes.^69^ The mitochondrial transcription mechanism, consisting of TFAM, TFB2M and mitochondrial RNA polymerase (POLRMT), initiates the expression of mtDNA.^70^ TFAM binds to mtDNA to change its structure. TFB2M is also encoded by nuclear DNA and is transported from the cytoplasm to mitochondria as a transcriptional factor of mitochondrial genes. In addition, TFB2M and POLRMT interact with TFAM to induce nucleus gene expression.^71^

Recent studies have shown that activation of the Nrf2 pathway enhances mitochondrial biogenesis. NRF‐2 binds specifically to cis elements in the promoter of cytochrome oxidase subunit IV (COXIV) to induce COXIV transcription.^72^ In addition, Nrf2 interacts with many other genes related to respiratory chain expression, including TFAM, TFB1M and TFB2M, to promote mitochondrial biogenesis.^73^, ^74^

In many cases, Nrf1 and Nrf2 have the same targeted genes. Nrf1 and Nrf2 both bind to the TFB1M and TFB2M promoters, as was detected by chromatin immunoprecipitation.^75^ Three of the four succinate dehydrogenase (complex II) subunit genes also have Nrf1/2 binding sites in their promoter.^76^ Piantadosi and his colleagues have shown that Nrf2 activation enhances mitochondrial biogenesis in cardiomyocytes via multiple Nrf2 binding sites in the Nrf1 promoter. Therefore, Nrf1 plays a major role in mitochondrial biogenesis. Like Nrf1, Nrf2 is involved in the regulation of the expression of essential respiratory chain proteins and key components of the mitochondrial transcription mechanism. This complex mechanism ensures the coordination among genes and promotes mitochondrial biogenesis.

### 4.3 Mitophagy pathway

During IRI, damage to mitochondria is further aggravated by oxidative stress. Therefore, it is vital to remove dysfunctional mitochondria. Mitophagy refers to the process of selective removal of redundant or damaged mitochondria by autophagy, and it plays a significant role in mitochondrial control and cell survival.^77^

The PTEN induced kinase 1 (PINK1)/ parkin RBR E3 ubiquitin protein ligase (Parkin)‐mediated signalling pathway is the best‐characterized mitophagy pathway.^78^ Upon oxidative stress, mitochondrial depolarization and a decrease in membrane potential occurs; PINK1 can no longer enter the mitochondria, and its protein hydrolysis, cleavage and subsequent degradation are inhibited. Furthermore, PINK1 accumulates in the depolarized mitochondrial outer membrane, where it is phosphorylated, and then recruits several other proteins, including Parkin and TANK‐binding kinase 1. After Parkin is activated by phosphorylation, mitophagy is induced by ubiquitination of voltage‐dependent anion channel (VDAC) and recruitment of the receptor protein sequestosome 1 (p62/SQSTM1).^79^ Phosphorylation of parkin is one of the key steps of mitophagy. Therefore, other potential phosphorylation sites can be found by bioinformatics, liquid chromatography‐mass spectrometry, and microarray. In addition, mitochondrial dynamics can also affect mitophagy. The size of mammalian autophagy bodies ranged from 500 nm to 1500 nm. Therefore, it can be predicted that mitochondrion division occurs before mitochondrial autophagy. Experimental evidence also confirmed that the expression of mitochondrion fission‐related factors or the degradation of mitochondrial fusion‐related factors is necessary to induce mitophagy. These factors regulate mitophagy by interacting with microtubule associated protein 1 light chain 3 alpha (LC3) adaptor protein or LC3 receptor. For example, dynamin 1 like (Drp1), as an important mitochondrial fission protein, can interact with LC3 receptors FUN14 domain containing 1 and BCL2 like 13 to induce mitophagy. In addition, optic atrophy 1 (OPA1), as a mitochondrial fusion protein, can also be used as an important factor to induce mitophagy.

PGC‐1α has been shown to participate in mitophagy,^80^ but the specific mechanism is still unclear. Both PGC‐1α and PINK1 are highly expressed in the brain,^81^ however, when the expression of PINK1 is knocked down, the PGC‐1α expression is also reduced.^82^ The exact interaction mechanism is unclear, but it is possible that PGC‐1α contributes to transcriptional regulation and activates the promoter region of PINK1. Down‐regulation of PINK1, a protein kinase, may in turn affect the phosphorylation of PGC‐1α and decrease its cytoplasmic stability. In addition, the theoretical downstream target genes of NRF‐1 include PINK1 and Parkin,^83^ hence we believed that PGC‐1α may regulate the expression of PINK1 and Parkin indirectly by regulating NRF‐1, and thus may participate in mitophagy. In addition, the p62/SQSTM1 promoter contains ARE elements, allowing NRF‐1 and −2 to bind and mediate transcription,^84^ and it has been found that NRF‐1 rapidly activates p62/SQSTM1 and induces autophagy under oxidative stress.^85^ NRF‐2 has also been shown to promote the expression of p62/SQSTM1 by interaction with the transcription co‐regulator SPBP.^86^ The STGE motif allows p62/SQSTM1 to bind and target kelch‐like ECH‐associated protein 1 (Keap1) for autophagic degradation, eliminating negative regulation, and thus promoting an accumulation of NRF‐2.^87^ Ser‐349 in the STGE motif of p62/SQSTM1 can be phosphorylated by mammalian target of rapamycin complex 1, which senses oxidative stress,^88^ and phosphorylation of Ser‐349 enhances its affinity for Keap1 binding.^89^ Keap1 removal results in an increase in NRF‐2 protein expression. p62/SQSTM1, the autophagic connector proteins CALCOCO‐2/NDP52, serine/threonine kinase ULK1, E3 ubiquitin ligase ATG5 and ubiquitin‐like modifier GABA Type A receptor‐related protein 1 all have been demonstrated to interact with NRF‐2.

## 5 CONCLUSIONS AND FUTURE PERSPECTIVES

Oxidative stress is the main factor leading to mitochondrial dysfunction. The damaged mitochondria are fed back to the nucleus through ‘retrograde regulation’. The nucleus resists mitochondrial damage by ‘anterograde regulation’. We propose the important role of mitonuclear communication mechanism in IRI. PGC‐1α can resist IRI‐induced mitochondrial damage by up‐regulating a series of mitochondrial related genes. PGC‐1α/NRF‐1/2 reduce the production of ROS caused by IRI via up‐regulation of antioxidant genes and furthermore eliminate damaged mitochondria by enhancing mitophagy. Additionally, PGC‐1α/NRF‐1/2 promotes the biogenesis of mitochondria in the myocardium. In addition, we believe that mitochondrial biogenesis can help mitophagy to produce healthy mitochondria. However, it is still unclear which of the three signalling pathways mediated by PGC‐1α/NRF‐1/2 play a major role in IRI. This may depend on whether the patient has other underlying diseases and the degree of blood supply. In conclusion, PGC‐1α may effectively resist IRI through ‘anterograde regulation’ of mitonuclear communication.

Although a large amount of evidence has elucidated the underlying mechanism of PGC‐1 α associated with heart disease, drug development for successful treatment of PGC‐1 α is still in its infancy. Therefore, we need to continue to explore the mechanism of PGC‐1 α in the heart. In addition, data on the pathological effects of PGC‐1 α on heart disease in rodents and humans are limited and often contradictory. Future research should focus on clarifying the signalling network of PGC‐1 α in IRI. In addition, there are many problems to be solved. For example, how does PGC‐1 α activity change in IRI? How to control PGC‐1 α signalling pathway to play a powerful role in height regulation without side effects? Therefore, it is necessary to have a more comprehensive and detailed understanding of the role of PGC‐1 α in heart disease before studying the therapy for PGC‐1 α signalling pathway.

## CONFLICT OF INTEREST

The authors declare no conflict of interest.

## AUTHOR CONTRIBUTIONS

**Yanqing Li:** Formal analysis (equal); Project administration (equal); Software (equal); Validation (equal); Writing‐original draft (equal). **Yan Jiao:** Data curation (equal); Methodology (equal); Visualization (equal); Writing‐original draft (equal); Writing‐review & editing (equal). **Ya‐Nan Liu:** Supervision (equal); Writing‐review & editing (equal). **Jiaying Fu:** Validation (equal). **Lian‐Kun Sun:** Conceptualization (equal); Funding acquisition (equal); Investigation (equal); Resources (equal); Writing‐original draft (equal). **Jing Su:** Conceptualization (equal); Funding acquisition (equal); Resources (equal); Validation (equal); Writing‐review & editing (equal).
